# Influence of endoleak positions on the pressure shielding ability of stent-graft after endovascular aneurysm repair (EVAR) of abdominal aortic aneurysm (AAA)

**DOI:** 10.1186/s12938-016-0249-z

**Published:** 2016-12-28

**Authors:** Jie Li, Xiaopeng Tian, Zhenze Wang, Jiang Xiong, Yubo Fan, Xiaoyan Deng, Anqiang Sun, Xiao Liu

**Affiliations:** 1 Key Laboratory for Biomechanics and Mechanobiology of Ministry of Education, School of Biological Science and Medical Engineering, Beihang University, Beijing, China; 2 National Research Center for Rehabilitation Technical Aids, Beijing, China; 3 Department of Vascular and Endovascular Surgery, The Chinese PLA General Hospital, Beijing, China

**Keywords:** Endoleak, Abdominal aortic aneurysm, Pressure shielding ability

## Abstract

**Background:**

Abdominal aortic aneurysm (AAA) is a kind of dangerous aortic vascular disease, which is characterized by abdominal aorta partial enlargement. At present, endovascular aneurysm repair (EVAR) is one of the main treatments of abdominal aortic aneurysm. However for some patients after EVAR the aneurysm re-expanded and even ruptured, leading to poor postoperative effect. The stent-graft endoleak after EVAR was realized to influence the AAA in-sac pressure and contribute to the aneurysm re-enlargement.

**Methods:**

In order to analyze the influence of endoleaks positions on the pressure shielding ability of stent-graft after EVAR, type I and type III endoleak models were reconstructed based on computed tomography (CT) scan images, and the hemodynamic environment in AAA was numerically simulated.

**Results:**

When the endoleak was at the proximal position the pressure shielding ability will be obviously weakened. While, the pressure shielding ability was higher in the systole phase than that in diastole phase when the endoleak located at the middle or distal positions. Unfortunately, when the endoleak located at the proximal position, the pressure shielding ability would be relatively weak in the whole cardiac cycle.

**Conclusions:**

The results revealed that the influence of endoleaks on pressure shielding ability of stent-graft was both location and time specific.

## Background

Abdominal aortic aneurysm (AAA) is local dilatation of the aortic wall. While when the abdominal aorta expands to about 50% larger than the normal size, it can be diagnosed as AAA [1]. Approximately 5~7% of the elderly population are at risk of developing AAAs, most of which are over the age of 60 [2]. In United States ruptured AAAs are the 13th leading cause of death and it’s mortality rate is up to 75% [3]. Due to the high morbidity and mortality of AAA, the pathogenesis and treatment have become a research hotspot in recent years.

Endovascular aneurysm repair (EVAR) is a mechanical solution to the AAAs expansion and rupture [4, 5]. It can effectively protect the aneurysm sac from high and pulsatile blood pressure in the abdominal aortic artery. For its advantage of slight trauma, rapid recovery, reduced wound infection rates, etc., EVAR has developed significantly in recent years [6, 7].

**Fig. 1:**
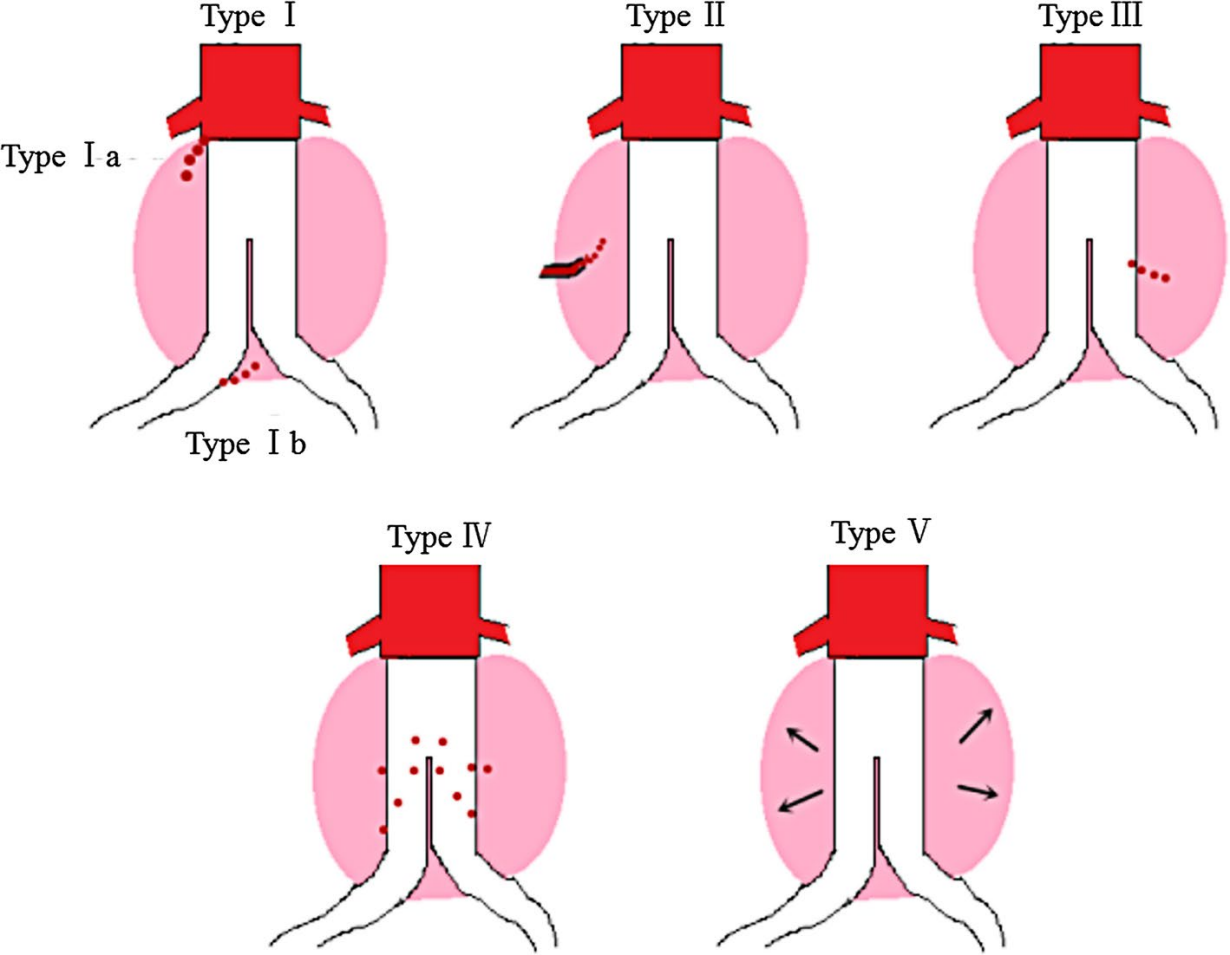
Five types of endoleaks

However, additional problems such as endoleak after EVAR have emerged with the widespread use of EVAR. Endoleak is defined as the occurrence of sustainable blood flow between the stent-graft (SG) and the aneurysm sac [8], which will directly weaken the pressure shielding ability of stent-graft. Endoleaks have been identified in 5.4 to 47.7% of the patients who underwent EVAR [9]. Endoleaks are divided into five types according to etiology [8]. Type I: These endoleaks are caused by leakage from the proximal anchoring (type Ia) or distal anchoring (type Ib). Type II: The aneurysm is further perfused via lateral branches (e.g. the lumbar artery or inferior mesenteric artery). Type III: The aneurysm sac is further perfused by the overlap zones of individual stent prosthesis components. Type IV: Blood can escape into the aneurysm sac through stent material. Type V: Checkups reveal an increase in aneurysm diameter, but no contrast substance can be detected outside the stent prosthesis. These five types of endoleaks are shown in Fig. 1.

Many clinical studies reported that endoleaks could induce high incidence of aneurysm re-enlargement and rupture after EVAR by persistent pressurization of the aneurysm sac [9–14]. Thus, the mechanism of AAA re-enlargement induced by endoleaks need to be fully understood, especially when EVAR is fairly common all over the world today. However, to authors acknowledge so far, less studies have quantitatively investigated the influence of endoleaks on the pressure change in the AAA sac. In the present study, type I and type III endoleaks models were reconstructed based on real patient medical images. Then the hemodynamic effects of type I and type III endoleaks were numerically simulated and discussed, especially the pressure change in the AAA sac.

## Methods

### Models

**Fig. 2:**
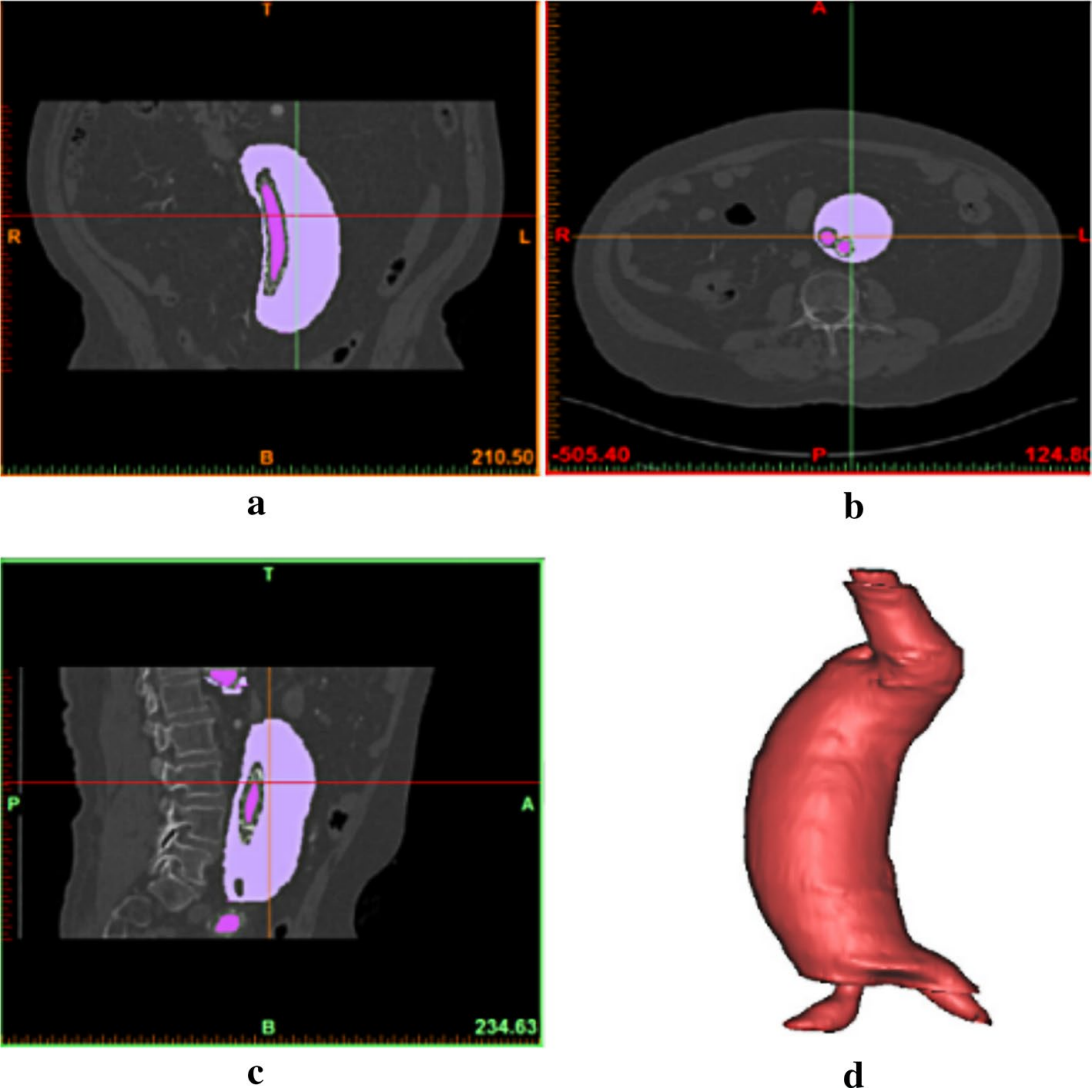
CT images and the reconstructed model with Mimics. **a** coronal view of CT images, **b** axial view of CT images, **c** sagittal view of CT images, **d** reconstructed model with Mimics

**Fig. 3:**
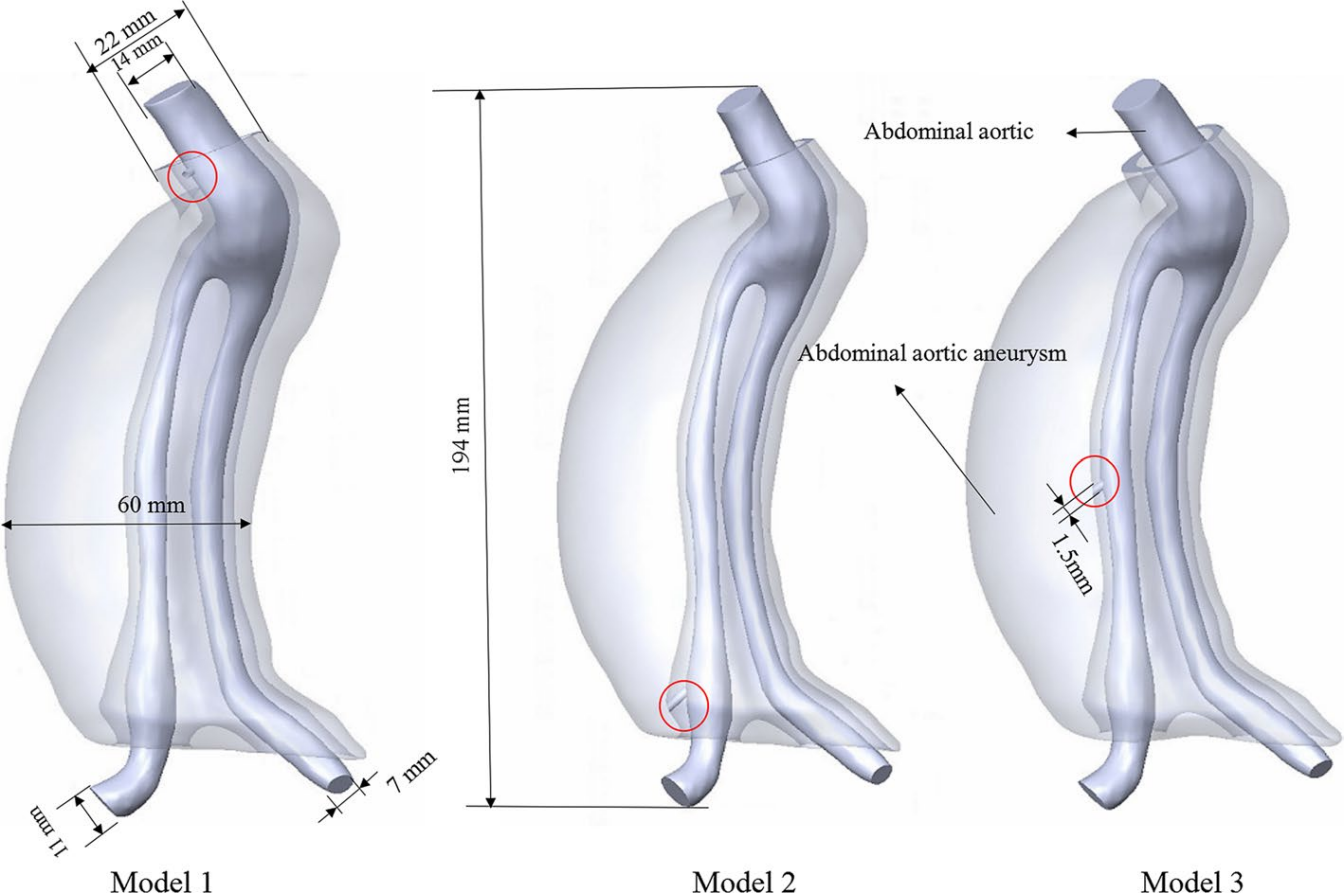
Endoleak models reconstructed from CT images. The cylindricals with diameter of about 1.5 mm were used to represent the leakage. Model 1 and model 2 presented type I endoleak with leakage at the proximal and distal anchoring positions respectively. Model 3 presented type III endoleak, with one leakage at the middle overlap zones. Red circles were used to mark different endoleak positions

Abdominal aortic aneurysm models after EVAR were reconstructed based on the computed tomography (CT) scan images obtained from Chinese PLA General Hospital (Beijing, China). The CT-relevant parameters were as follows: 0.5 mm slice thickness, 1.5 mm reconstruction spacing/increment, 0.5 mm slice overlap and a 512 × 512 image resolution. Mimics (v9.0, Materialise, Ann Arbor, MI, USA) were used to reconstruct models bases on the CT images. Some CT images and the reconstructed model with Mimics software to better present the geometry of the model are shown in Fig. 2. The reconstructed abdominal aortic aneurysm models from Mimics were smoothed by Rapidform (v2004, INUS, Korea) and modified by Geomagic (Geomagic Studio, Geomagic, USA), Solidworks (Solidworks Corporation, Boston, MA, USA) to mimic endoleaks geometry. Simplified Type I endoleak models (model 1 with one leakage from the proximal anchoring and model 2 with one leakage from the distal anchoring) and type III endoleak model (model 3 with one leakage from the middle overlap zones) were built as shown in Fig. 3.

### Mesh generation

**Fig. 4:**
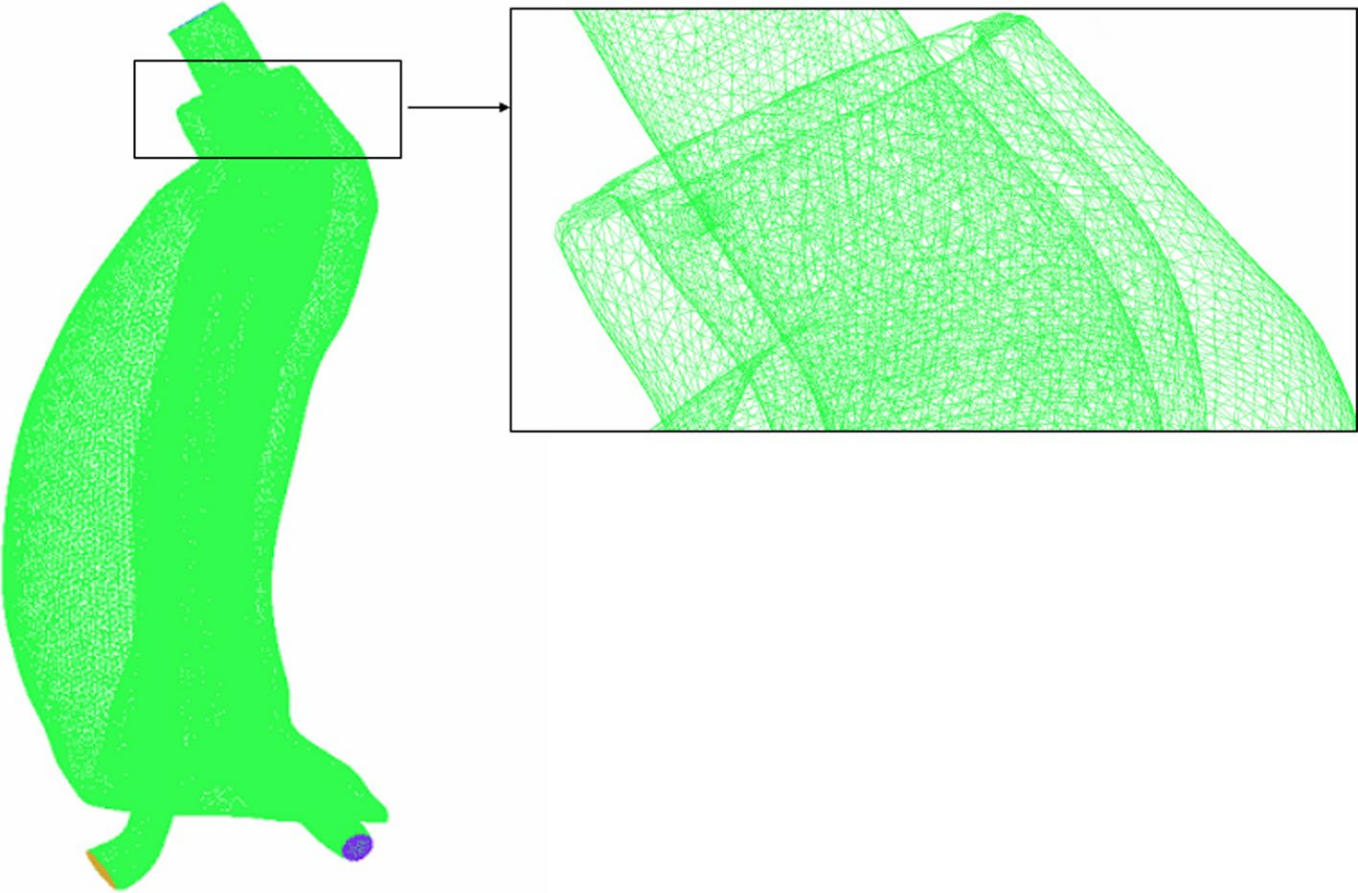
Meshes of the endoleak models. Models were meshed with mixed tetrahedral and hexahedral volume elements. The minimum and maximum size of elements were 0.2 and 1 mm respectively

ICEM (ANSYS, Inc., Canonsburg, PA, USA) were used to generate a mixture of tetrahedral and hexahedral elements meshes of these models (Fig. 4). The maximum and minimum sizes of the mesh were set to 1.0 and 0.2 mm respectively. To improve mesh generation quality and guarantee the accuracy of computation results, the number of the boundary layer was set to 5, the height ratio was set to 1.3, and the total height was set to 0.2 mm, as the same methods used in our previous study [15]. For type I, the node numbers were 651,116 and 651,476 for the model 1 and model 2, respectively. For type III, the node number is 651,713.

### Boundary conditions

**Fig. 5:**
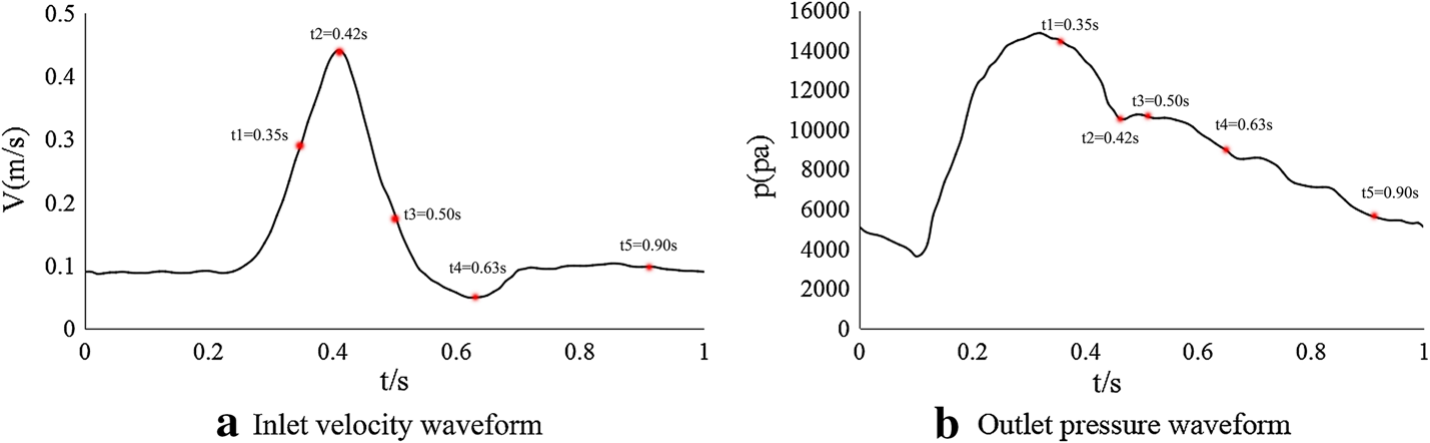
Boundary conditions. **a** Inlet velocity waveform **b** Outlet pressure waveform

To describe the characteristics of the detailed hemodynamics in the AAA, unsteady blood flow was simulated in the reconstructed models by software Fluent (Fluent Inc., Lebanon, NH, USA). A periodic velocity curves derived from the studies conducted by Walsh PW [16] and Z. Li [3] showed inlet flow rate was set as inlet boundary condition, and a periodic outlet back pressure was set as the outlet boundary condition. Figure 5a [17] shows inlet pulsatile flow wave. The outlet pressure wave was shown in Fig. 5b [17].

To quantitatively provide hemodynamic results of the models, the SIMPLE algorithm was applied, and a segregated algorithm was applied to solve all equations, with the application of the necessary physiological boundary conditions.

### Assumptions and governing equations

The blood vessel was assumed as rigid and impermeable wall. Blood was modeled as Newtonian, homogeneous and incompressible [18, 19] fluid. The numerical simulation was conducted based on a three-dimensional incompressible Navier–Stokes equation and the conservation of mass:

1 $$\rho \left[ {\frac{{\partial \overset{\lower0.5em\hbox{$\smash{\scriptscriptstyle\rightharpoonup}$}} {u} }}{\partial t} + \left( {\overset{\lower0.5em\hbox{$\smash{\scriptscriptstyle\rightharpoonup}$}} {u} \cdot \nabla } \right)\overset{\lower0.5em\hbox{$\smash{\scriptscriptstyle\rightharpoonup}$}} {u} } \right] + \nabla p - \nabla \cdot \tau = 0$$

2 $$\nabla \cdot \overset{\lower0.5em\hbox{$\smash{\scriptscriptstyle\rightharpoonup}$}} {u} = 0$$

The fluid velocity vector and the pressure was represented by $$\overset{\lower0.5em\hbox{$\smash{\scriptscriptstyle\rightharpoonup}$}}{\text{u}}$$ and *p* respectively. ρ and μ are the density and viscosity of blood (μ = 3.5 × 10^−3^ kg/m s and ρ = 1050 kg/m^3^), and τ is stress tensor. The CFD software package, ANSYS Fluent 15.0 (ANSYS, Lebanon, NH, USA) was used for the simulations. Blood flow has been found to be laminar in AAA, even during exercise [20]. The convergence criterion was set to 1 × 10^−5^. Six cycles were required to obtain a convergence for the transient analysis, with 100 steps in each cycles (T = 1 s). As the same settings used in previous study [15].

## Results

In this study we focused on the hemodynamic effects of endoleaks in abdominal aortic aneurysm, especially the detailed parameters related to the total pressure.

### Pressure contours

**Fig. 6:**
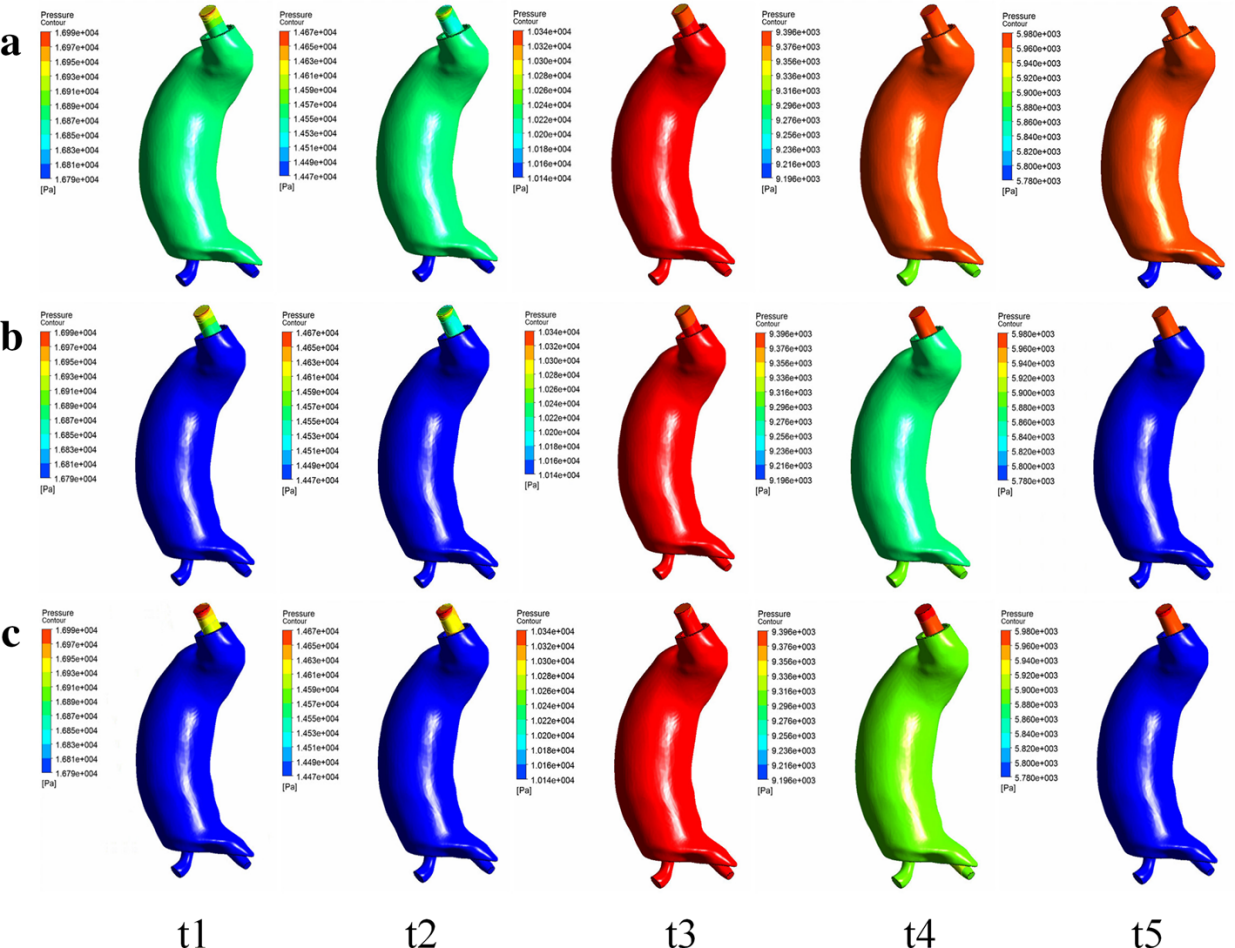
Panel **a** showed the contours of the total pressure at t1 t2 t3 t4 and t5 of model 1. Similarly panel **b** and panel **c** showed the contours of model 2 and model 3 respectively

Five time points from one cardiac cycle, early systole (t1), peak systole (t2), early diastole(t3), nadir diastole (t4), and later diastole (t5), were defined and shown in Fig. 5a. The hemodynamic results at these five time points were presented in Fig. 6.

Figure 6 showed the contours of the total pressure of model 1 (type Ia endoleak with one leakage from the proximal anchoring), model 2 (type Ib endoleak with one leakage from the distal anchoring), and model 3 (type III endoleak with one leakage from the middle overlap zones), respectively. In order to compare the different models, in these contours we chose the inlet average pressure as upper limit and unified the range for 200 Pa. Pressure contours of model 1 in Fig. 6a showed that the pressure on the AAA wall was similar to that in the aorta at these five selected time phases. Pressure contours of model 2 in Fig. 6b showed that at t1, t2, t4 and t5 the pressure of AAA wall was lower than that in the aorta, while at t3 the pressure difference was quite modest. Pressure contours of model 3 in Fig. 6c also showed that at t1 t2 t4 and t5 the pressure of AAA wall was lower than that in the aorta, while at t3 the pressure difference was quite modest too.

### Histogram

**Fig. 7:**
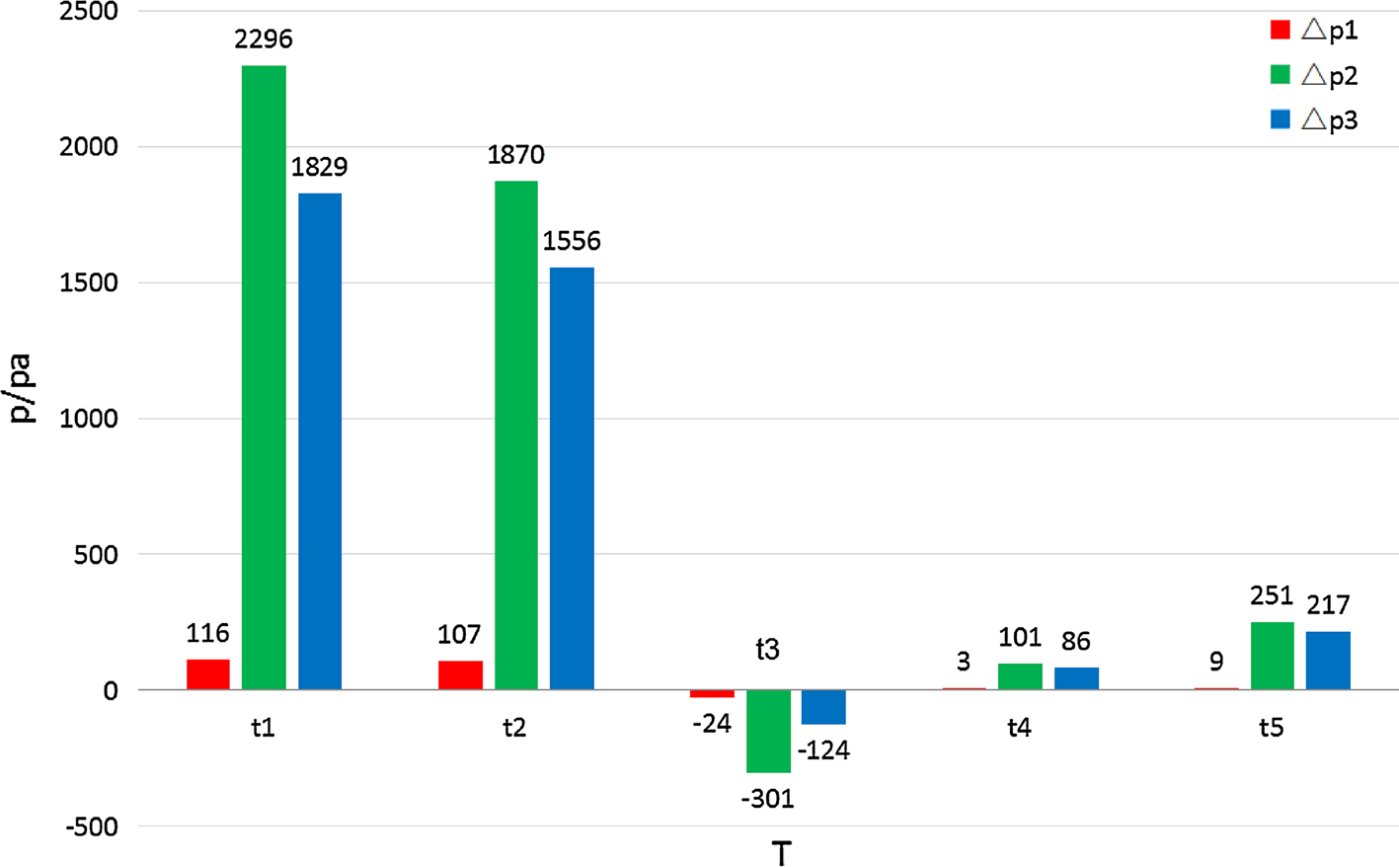
Average pressure difference between inlet and AAA wall of three models (Δp = P_Inlet_ − P_AAA wall_)

To quantitatively compare pressure features of different models, the histograms of the average pressure difference between inlet (P_inlet_) and AAA wall (P_AAA wall_) were calculated and shown in Fig. 7. The Δp1, Δp2, and Δp3 represented the average pressure difference of the model 1, model 2, and model 3 respectively. The histograms indicated the same tendency as above mentioned contours. Δp2 and Δp3 are significantly higher than Δp1, especially at t1 and t2 time phases. At t3 the pressure differences for all the three models were negative values, which indicated that the pressure in the sac was higher than that in the aortic inlet.

## Discussion

A variety of clinical and experimental studies have shown that endoleaks would cause the pressure increase in AAA sac and consequently induce AAA re-enlargement and rupture [21, 22]. Thus, quantitatively identifying the influence of endoleaks on the pressure change of AAA [23], could help us know more about the mechanism of AAA re-enlargement after EVAR and give advice for clinical treatment and device design.

In this study, 3-D models of different types of endoleaks based on medical CT images were reconstructed and the hemodynamic effects of endoleaks were numerically simulated. Simulation results revealed two important conclusions: Firstly, the pressure difference between the aortic inlet and the AAA wall was sensitive to the endoleak positions. When the endoleak was near the proximal position (Fig. 6a, model 1), the pressure of AAA was similar to that at the aortic inlet. While when the endoleaks were near the distal or middle positions (Fig. 6b, c, model 2, 3), the pressure of AAA was lower than that at the aortic inlet. Secondly, the pressure difference between the aortic inlet and the AAA wall was sensitive to time phases. The influence of endoleak positions on pressure difference was more significant in systole phase (t1, t2) than that in diastole phase when the endoleak was at the middle and distal positions (Type III and Type Ib). At time t3, the pressure in the AAA was slightly higher than that at the aortic inlet, which might due to the ending phase of systole. Because at t3, t4 and t5, the pressure both at the aortic inlet and in the AAA was low, the pressure differences at these three time phases would have limited influence on the AAA re-enlargement.

As the pressure difference between the aorta and AAA sac is in proportion to the pressure shielding ability of stent-graft, the present study revealed that the influence of endoleak on pressure shielding ability of stent-graft was both location and time specific. When the endoleak was at the proximal position the pressure shielding ability will be obviously weakened. While the pressure shielding ability was higher in the systole phase than that in diastole phase, when the endoleak located at the middle or distal positions. Unfortunately, when the endoleak located at the proximal position, the pressure shielding ability would be very weak in the whole cardiac cycle, which alerted us that the endoleak at the proximal positions would be more dangerous to the AAA stability.

As Siem A. Dingemans [9] mentioned, aneurysm enlargement after EVAR remains a subject of debate and various types of endoleaks require individual treatment approach. The present study took type I and type III endoleaks as examples and numerically simulated their pressure features in the AAA, which would help us identify the influence of endoleaks on pressure shielding effect of stent-graft. As a preliminary study, only numerical simulation has been conducted to investigate flow features in AAA models after EVAR. In order to guarantee the results’ validity, small size meshes, thin boundary layers were used in model meshing. Besides, proper boundary conditions and assumptions, strict iteration convergence criterion, etc. were all helpful to improve the simulation accuracy. To further prove the validity of this study, in vitro and in vivo experiments will be conducted in authors future study. Also in the future, more detailed simulations and more endoleak types are expected to be considered, to help fully understand the mechanism of AAA re-enlargement after EVAR and give references and suggestions to AAA treatment and device design.

## Conclusions

In our study, the results revealed that the influence of endoleak on pressure shielding ability of stent-graft was both location and time specific. More detailed simulations and more endoleaks types are needed to continue our investigation.
